# Supplementary material for: Are carbon emissions trading and green financial instruments synergistic? -Comprehensive quantitative research based on content analysis
Source: PLoS One. 2024 Mar 7;19(3):e0298601. doi: 10.1371/journal.pone.0298601 (PMC10919601; doi:10.1371/journal.pone.0298601)
Supplement: S1 Data — (ZIP) [file pone.0298601.s001.zip › Data/地级市Z_F工作报告.zip!/╕╩╦α/┬ñ─╧╩╨/└┤╫╘╣1⁄2╓┌║┼í╛┬φ┐╦╩2╛▌═°í┐_2016.docx]

各位代表：

　　现在，我代表市人民政府向大会报告工作，请予审议，并请各位政协委员和列席人员提出意见。

　　“十二五”规划完成情况和2015年工作回顾

　　“十二五”时期，是我市抢抓历史机遇、夯实发展基础、实现重点突破、推进转型跨越发展的重要时期。五年来，在市委的坚强领导下，我们深入贯彻党的十八大，十八届三中、四中、五中全会和习总书记系列重要讲话精神，全面落实省委、省政府一系列重大决策部署，深化实施“433”发展战略，攻坚克难，开拓进取，苦干实干，奋力推进全市经济社会持续健康发展，圆满完成了“十二五”规划主要目标任务。

　　——经济运行稳中有升。我们牢牢把握稳中求进总基调，主动适应经济发展新常态，统筹推进稳增长、促改革、调结构、惠民生、防风险各项工作，经济发展质量效益同步提升，主要经济指标保持了持续快速增长。“十二五”末，全市生产总值完成315亿元，比“十一五”末增长69.6%，年均增长11.2%。一是农业经济稳步发展。加快推进农业特色产业提质增效，基地面积达到1089万亩，完成核桃、花椒、油橄榄高接换优1000万株，培育发展农民专合组织4970家、市级以上龙头企业169户，完成“三品一标”认证226个，农特产品品牌建设成效显著，连续4年成功举办农特产品产销对接洽谈会，农业产业化经营水平得到提升；新建标准化规模养殖场（小区）1708个，规模化养殖比重达到40%，比“十一五”末增长7%。积极落实科技增粮措施，粮食总产实现“九连增”。农村居民人均可支配收入完成5405元，比“十一五”末增长97.3%，年均增长14.6%。二是工业经济总量不断扩大。积极培育以有色冶金、白酒酿造、农特产品加工、建筑建材、水电能源、现代制药为重点的优势主导产业，工业经济总量进一步扩大，全市规上工业增加值完成41.2亿元，比“十一五”末增长1.04倍，年均增长15.3%。中小微企业达到3864家，非公经济增加值完成155亿元，占全市GDP总量的50%以上。实施市列重点工业项目99个，建成投产50个。园区建设进一步加快，入园企业达到131户。三是第三产业发展水平不断提升。建成了成县电子商务产业孵化园、陇南特色农产品交易市场、哈达铺华昌药材城等一批物流仓储、专业市场。电子商务实现突破性发展，开办网店8674家，实现销售34亿元，带动就业5.4万人。文化旅游融合发展迈出坚实步伐，连续成功举办乞巧女儿节、白马人民俗文化旅游节等系列文化旅游节会，省级、国家级非物质文化遗产申报工作和古建筑、古民居保护开发取得积极进展。重点旅游景区建设成效显著，全市4A级景区达到9家，乡村旅游、中医养生度假等产业起步良好。全市社会消费品零售总额完成90.8亿元，比“十一五”末增长95.7%，年均增长14.4%。文化产业增加值完成5.71亿元。城镇居民人均可支配收入完成18915元，比“十一五”末增长74.4%，年均增长11.8%。产业结构趋于基本合理，三次产业结构由“十一五”末的26.3：28.6：45.1调整为“十二五”末的22.31：23.14：54.55。

　　——改革开放持续深化。政务服务中心规范化建设全面推进，新建了市区两级政务服务大厅，全市九县区都成立了政务服务中心，90%以上乡镇建立了便民服务大厅。“五张清单一张网”建设如期完成。工商、质监、卫生计生、食品药品监管等机构改革任务顺利完成。公共资源交易管理和平台建设得到加强，资源配置市场化程度得到较大提升。财税体制改革加快推进，国库集中支付、预算管理改革和国资国企改革深入实施。医药卫生体制改革进展良好，户籍制度改革稳步实施。积极推进向南开放，精心谋划打造兰（成）渝经济带陇南黄金段，加快融入长江经济带、成渝经济圈和大九寨旅游经济圈，先后与绵阳、广元、汉中、宝鸡等市签订了战略合作框架协议，在打造毗邻地区开放开发共同体方面迈出了坚实步伐。招商引资取得显著成绩，项目落地率和资金到位率逐年稳步提升。

　　——扶贫攻坚取得阶段性成效。坚持把扶贫开发作为全局工作的重中之重，全面落实“1236”扶贫攻坚行动，紧盯25个特困片区，坚持资金向片区倾斜、项目向片区整合，领导和帮扶力量向片区汇聚，形成了合力攻坚的良好格局。围绕贯彻落实省委、省政府“1+17”精准扶贫方案，制定出台一系列政策措施，按照“853”挂图作战的要求，创新精准扶贫方式，打响了精准扶贫精准脱贫攻坚大决战。贫困乡村路、水、电、讯、房等基础设施条件得到较大改善，建成通村硬化路9271公里，通畅率达到89.1%，解决了100.9万农村人口安全饮水问题，贫困村动力电覆盖率达到94%，实施整村推进项目750个、完成易地扶贫搬迁1.15万户5.09万人。全市特色产业总产值达到150亿元，人均特色产业收入2800元以上。累计输转农村劳动力428.5万人次，创收494.7亿元，年均增长14.8%。双联与扶贫深度融合，有效发挥了双联助推作用。电商扶贫创出了“陇南模式”，我市被国务院扶贫办确定为全国电商扶贫试点市，荣获2015中国消除贫困创新奖。全市贫困人口下降到50万人，年均减少贫困人口近20万人，贫困发生率下降到20.4%，下降33个百分点。

　　——城乡一体化建设步伐加快。紧紧围绕打造“宜居宜游宜业山水园林城市”目标，进一步优化区域布局，稳步推进城乡一体化建设，城镇化率达到28.16%，比“十一五”末提高8.46个百分点。市区和八县县城新区开发、旧城改造取得阶段性进展，武都中心城市建设步伐加快，城市道路、供排水、集中供热供气、垃圾污水处理等市政基础设施项目加快实施，城市品位和综合服务功能进一步提升。礼县、康县及成县小川、宕昌县哈达铺等省级新型城镇化试点和康县多规合一试点进展良好，小城镇综合改革试点工作稳步推进，政府职能转变、户籍制度、投融资体制、土地利用等综合配套改革取得积极进展。建成生态文明新农村1668个，占行政村总数的52.1%，建成省级“美丽乡村”18个，农村人居环境进一步改善。

　　——基础设施建设强力推进。抢抓政策机遇，全力推进“3341”项目工程，为拉动全市经济持续增长发挥了重要支撑作用。通过灾后重建，全市城乡面貌发生了历史性变化。着力破解交通瓶颈制约，加快构建铁路、高速公路、航空三位一体的交通网络，兰渝铁路加快推进，成州机场全面开工，徽两高速、渭武高速试验段开工建设，武九高速前期工作进展顺利，武罐、成武、十天高速公路建成通车，实现了高速公路从无到有的历史性突破，达到380公里；国省干道、县乡公路全面升级改造，综合交通网络逐步形成。水利、电网和信息化建设取得显著成效。智慧城市建设进展良好，陇南市大数据政务中心建成投用。五年累计完成固定资产投资2197.8亿元，年均增长26.9%，是“十一五”期间的3.4倍。

　　——生态环境逐步改善。生态文明示范市建设、“两江一水”综合治理规划和水生态文明城市建设试点顺利实施，退耕还林、天然林保护、长防林公益林建设等重点生态工程统筹推进，森林覆盖率达到40.4%。江河干支流、中小河流治理取得积极成效。污染减排取得新进展，水、大气污染治理成效显著，矿山、尾矿库专项整治持续开展，主要污染物总量减排在省控指标以内。农村环境整治取得明显成效。积极创建国家级地质灾害综合防治试验区，建成陇南市自然灾害监测预警指挥系统和市级水资源实时监控与管理信息系统，新一代天气雷达系统开始试用，防灾减灾能力进一步提升。

　　——财政金融支撑能力显著增强。“十二五”末，全市大口径财政收入和一般公共预算收入分别完成51.5亿元、25.5亿元，比“十一五”末分别增长1.4倍、1.7倍，年均分别增长20%、22%。五年累计实现财政支出777.6亿元，年均增长7.35%，有力保障了重大项目、产业培育和改善民生等各项重点工作。实施“3+1”金融体系建设，农村产权抵押贷款试点工作取得积极成效，成立陇南龙江城乡发展有限公司、陇南信通城市经济发展有限公司、陇南龙江资金池管理有限公司，强化政府投融资平台建设，有效缓解了农民、中小微企业和项目建设融资难问题。金融服务体系不断完善，直接融资取得突破性进展。扶贫互助资金项目进展良好。棚户区改造贷款争取工作成效显著。各类惠民信贷政策得到落实。全市信贷投放总量快速增长，存贷比达到65.2%，比“十一五”提高22.5个百分点，金融支撑能力明显提升。

　　———社会建设成效明显。坚持教育优先发展战略，各级各类教育协调发展，城乡办学条件持续改善，教师队伍不断加强，教育质量明显提升，市一中成功创建省级示范性高中，陇南师专三校合一顺利完成，陇南师专升本和陇南卫生职业技术学院创建工作稳步推进。科技事业得到较快发展，科技创新能力明显增强。卫生基础设施、基层卫生条件和公共卫生服务水平不断提升，全民医保体系基本健全，中医药事业和健康服务业加快发展。公共文化服务体系不断完善，一批文化、广电、体育基础设施建成投用，乡镇综合文化站、农家书屋、广播电视“村村通”实现全覆盖。社会保障和救助水平得到提高，城乡低保标准逐年提升，城乡居民全部纳入社会养老保险范围，养老服务体系日趋完善。就业创业扶持政策全面落实，大学生就业服务工作取得实效。保障性住房建设和城市棚户区改造取得显著成绩。坚持每年办成一批民生实事，解决了群众关心期盼的热点难点问题。安全生产责任和监管措施得到落实，食品药品安全监管全面加强。扎实开展社会稳定风险评估，深入推进社会治理创新，在全省率先开展农村社区建设试点，矛盾纠纷排查调处和信访工作深入开展，“六五”普法规划全面实施，平安陇南建设取得积极成效。民族宗教、外事侨务、统计、审计、档案、地方志、气象、地震、“双拥”、人防、民兵预备役和妇女儿童、残疾人、老龄、慈善、红十字会等各项事业取得新进步。

　　———政府自身建设得到加强。加快转变政府职能，大力推进简政放权，行政审批制度改革深入实施，政府效能得到提升。依法全面履行政府职责，深入推进政务公开和各领域办事公开，自觉接受人大法律监督和政协民主监督，建议提案办理工作取得实效。提请市人大常委会审议通过了《陇南市水能资源开发利用管理办法》和《陇南市矿业权审查审批管理办法》。严格落实党风廉政建设主体责任，认真贯彻落实中央八项规定、国务院约法三章和省委、市委各项规定，健全完善“三公”经费管理、预算和支出审批制度，全市“三公”经费开支明显下降。加强审计监督和行政效能监察，支持执纪执法机关查处了一批违纪违法案件。认真开展了党的群众路线教育实践活动和“三严三实”专题教育，着力解决政府班子和领导干部作风方面的突出问题，政府系统作风建设取得新成效。

　　各位代表，2015年，我们认真贯彻落实省委、省政府和市委决策部署，克服经济下行压力持续加大等不利因素，全力以赴推进全市经济社会持续快速发展，基本完成了市三届五次人代会确定的主要目标任务，生产总值增速居全省第一，其中农业增加值增速居全省第二、第二产业增加值增速居全省第三、第三产业增加值增速居全省第四，规模以上工业增加值增速居全省第三，建筑业增加值增速居全省第一，社会消费品零售总额增速居全省第二，农村居民人均可支配收入增速居全省第一。精准扶贫各项重点工作全面推进。完成了1365个贫困村和64.37万贫困人口建档立卡工作，精准扶贫大数据平台搭建运行。发放精准扶贫专项贷款23.3亿元。整合各类资金53亿元，片区攻坚取得新进展。贫困乡村基础设施建设加快推进。双联工作取得新成效。全市减少贫困人口14万人。农业特色产业提质增效年活动扎实开展。新增经济林面积27.5万亩，完成303.6万株核桃、12.8万株油橄榄高接换优任务。中药材标准化种植、苹果矮砧密植示范基地建设和生态放养鸡产业进展良好。新培育认定省级龙头企业6户。工业经济稳步增长。实施市列重点工业项目25个，郭家沟铅锌采选生产线、金徽优质酒酿造技术改造项目建成试产，佛仁制药二期扩建、青峰石材扩建等项目进展顺利。祥宇油橄榄产业园、康县5万吨核桃乳生产线等一批农特产品精深加工项目建成投产。规模以上工业增加值增长9.9%，工业用电量增长10%。第三产业快速发展。着力推动电子商务集中突破，取得了明显成效。文化旅游产业发展步伐加快，宕昌官鹅沟景区建设顺利推进，礼县大堡子山国家考古遗址公园、两当兵变红色文化园等重点项目进展良好，五阳路亚热带生态文化旅游圈建设启动实施。“十村百户千床”示范工程取得积极进展。”3341”项目工程全面推进。全市谋划实施投资500万元以上项目1197个，完成投资599.9亿元，争取国家、省上项目资金1769个40.3亿元。全年签约招商引资项目238个，签约资金539亿元，到位资金403.8亿元，同比增长31%。城乡建设取得新成效。全市实施市政基础设施项目111个，总投资30.5亿元，完成投资13.5亿元。房地产市场平稳健康发展，有力拉动了城市基础设施建设，城市管理和服务水平进一步提升。新建生态文明新农村417个、美丽乡村示范村38个。财政金融实现新突破。政府债券融资取得积极成效。“3+1”金融体系建设加快推进，农村产权确权抵押交易服务平台顺利建成并上线运行，为农业发展架通了融资桥梁。全市金融机构新增贷款余额102.3亿元，同比增长46%。社会事业和民生建设协调发展。全面改善贫困地区义务教育薄弱学校基本办学条件，农村幼儿园建设得到加快。建成乡村舞台826个，实施6个乡镇体育惠民工程。城乡低保规范清理工作得到加强。25件省列民生实事、10件市列民生实事全面完成。人民群众获得感和幸福感进一步提升。

　　各位代表，过去五年，面对经济发展进入新常态、下行压力持续加大和投资力度减弱等各种困难和挑战，我们认真贯彻落实中央“四个全面”战略布局和“五大发展”理念，以及省委、省政府和市委一系列重大决策部署，立足陇南实际，坚持有所为有所不为，扬长避短、后发赶超，深化实施“433”发展战略，开创了全市经济社会发展的新局面。取得这样的成绩实属不易，这是省委、省政府关怀支持和市委正确领导的结果，是市人大、市政协和社会各界监督支持的结果，是全市人民共同奋斗、开拓进取的结果。在此，我代表市人民政府，向奋战在各条战线上的广大干部群众，向驻陇南人民解放军、武警官兵和中央、省驻陇南单位，向各民主党派、工商联、无党派爱国人士、离退休老干部，向在陇南奋战的重大建设项目施工人员，向所有关心支持陇南经济社会发展的各界人士表示衷心的感谢和崇高的敬意！

　　各位代表，回顾过去五年的工作，我们也清醒地认识到，受历史、自然等多重因素的制约，全市经济社会发展仍然面临许多困难和挑战。主要表现在：贫困面大，贫困程度深，脱贫攻坚任务仍然十分艰巨；经济总量小，自我发展能力弱，经济结构性矛盾仍然突出；公共服务保障能力不强，基础设施还需要下更大气力提升完善，城乡居民持续增收的难度仍然很大；个别部门服务意识不强、办事效率不高、部分干部不作为的问题仍然存在。这些突出的矛盾和问题，在不断地提醒和警示我们，在今后的工作当中，要更加注重贯彻创新、协调、绿色、开放、共享“五大发展”理念，毫不动摇地深化实施“433”发展战略；更加注重经济发展的总体质量和整体水平，毫不动摇地推进结构调整和方式转变；更加注重人们的生存环境和生活质量，毫不动摇地抓好生态建设和环境保护；更加注重扶贫的实际效果和阶段目标，毫不动摇地打好精准扶贫精准脱贫攻坚战；更加注重当前的民生问题和社会稳定，毫不动摇地加强社会建设和法治建设；更加注重行政效率和政府形象，毫不动摇地转变政府职能和干部作风。

　　“十三五”时期全市经济社会发展的总体构想

　　“十三五”时期是我市深入贯彻“四个全面”战略布局和“五大发展”理念，深度落实省委、省政府决策部署，深化实施“433”发展战略，打赢脱贫攻坚战，与全国全省一道全面建成小康社会的关键时期。市委、市政府对编制好“十三五”发展规划高度重视，市委关于制定国民经济和社会发展第十三个五年规划的《建议》，提出了未来五年全市经济社会发展的指导思想、奋斗目标、战略重点和主要任务。根据市委《建议》精神，市政府编制了《国民经济和社会发展第十三个五年规划纲要（草案）》，已提请大会审议。下面，我就《纲要（草案）》作几点说明。

　　一、关于“十三五”时期经济社会发展形势的基本判断

　　综合分析判断国际国内和我市经济社会发展形势，“十三五”时期，我们面临着诸多重大战略机遇和有利条件。从政策机遇看，在国家实施“一带一路”战略这个最大机遇的牵引下，我市所面临的国家政策机遇、产业转移机遇、市场发展机遇正在加速释放，特别是中央出台《关于打赢脱贫攻坚战的决定》，国家不断加大对中西部地区基础设施建设支持力度，以及支持甘肃经济社会发展等重大决策部署，全省“3341”项目工程、“1236”扶贫攻坚行动、“6873”交通突破行动、“1+17”精准扶贫政策措施深入实施，为我市经济社会发展带来了难得的历史机遇，必将有力加快陇南扶贫攻坚、基础建设、资源开发、产业培育等各项事业发展。从发展条件看，我市地处甘、陕、川三省交汇的“金三角”地带，南倚成渝经济圈，东连关天经济区，北靠兰州新区，西接大九寨旅游经济圈，地处三大经济区的几何中心和旅游经济圈的重要结点上。　　　　　　随着铁路、高速、机场等重大交通项目的密集实施，陇南必将建成南下川渝、东进陕西、北上兰州、西连九寨的综合交通运输体系，成为连接兰白、对接成渝、衔接关天三大经济区的战略通道，区位优势将更加凸显，成为甘肃向南开放的桥头堡。从发展基础看，全市经济社会发展特别是扶贫攻坚、产业培育、基础设施、改善民生已经取得的成就，为实现整体脱贫和全面小康目标奠定了坚实基础。当前，全市广大干部群众脱贫攻坚奔小康的信心更加坚定，加快发展的氛围更加浓厚。只要我们团结一心，鼓足干劲，拼搏进取，一定能够如期实现整体脱贫和全面小康目标。

　　二、关于“十三五”发展的指导思想和预期目标

　　“十三五”时期，全市经济社会发展的指导思想是：高举中国特色社会主义伟大旗帜，全面贯彻落实党的十八大，十八届三中、四中、五中全会和习近平总书记系列重要讲话精神，认真落实省委十二届十四次全委会、市委三届十一次全委扩大会议精神，牢牢扭住“四个全面”战略布局，坚持“五大发展”理念和“八个并重”导向，深化实施“433”发展战略，坚决打赢精准脱贫攻坚战，统筹推进经济、政治、文化、社会、生态文明和党的建设，加快建设幸福美好新陇南，努力到2020年与全省全国一道全面建成小康社会。

　　预期目标是：生产总值年均增长8%左右。第一、二、三次产业增加值占生产总值的比重由2015年的22.3∶23.2∶54.5，调整到2020年的19∶25∶56。固定资产投资总额年均增长10%。工业增加值年均增长8%。一般公共预算收入与全市地区生产总值增长水平相适应。社会消费品零售总额年均增长9%。农村居民人均可支配收入和城镇居民人均可支配收入年均增长12%和8%。城镇登记失业率控制在4%以内。城镇化率达到35%以上。文化产业增加值占GDP比重达到5%。完成省上下达的节能减排控制指标。综合考虑各方面因素，实现这些目标是完全可能的。这里，需要着重说明的是，把经济增长速度确定为8%左右，主要考虑是，要如期实现整体脱贫、全面建设小康社会的目标，必须有一定的发展速度来支撑，否则，我们就难以在短期内缩小与全国全省的差距。把城乡居民收入年均增速分别确定为8%和12%，主要考虑是，保障和改善民生是经济发展的最终目的，必须着力推动城乡居民收入持续稳步增长，同时，按照实现整体脱贫和2020年全面建成小康社会的目标要求，城乡居民收入必须保持较高的增长速度。

　　三、“十三五”时期全市经济社会发展的重点任务

　　《纲要（草案）》在全面总结“十二五”规划经验，深入分析和把握陇南发展的阶段性特征的基础上，立足于打赢脱贫攻坚战、全面建成小康社会，立足于适应经济发展新常态、提高发展质量效益，立足于把握重大发展机遇、弥补发展短板，立足于贯彻“五大发展”理念、深化实施“433”发展战略，对“十三五”时期全市经济建设、文化建设、社会发展、生态文明建设、改革开放等方面作出了全面部署。重点抓好以下十个方面的工作：

　　1.坚持规模效益并重，促进经济稳增快转。一是做大做强农业特色产业。加快发展核桃、花椒、油橄榄、中药材等长效性特色产业和生态养殖、苹果、蔬菜、茶叶、马铃薯等区域性优势产业，做优食用菌、水产、蚕桑、苗木、魔芋等地方性特色产品，在提质增效上不断取得新突破。大力扶持培育龙头企业、家庭农场、农民合作社等农业新型经营主体。加强地理标志产品保护工作，强化农产品质量安全监管，把我市建成全国农产品质量安全监管示范市。加大中低产田改造和高标准农田建设力度，稳步提高粮食生产能力和综合保障能力。二是推动园区经济集约发展。鼓励引进先进生产技术与经营管理理念，加快铅锌、黄金等资源型产业转型升级，研发生产附加值较高的新型材料和精深加工产品。加快特色农产品深加工企业技术改造，加强与国内大型企业的战略合作，开发生产科技含量高、市场前景好的产品。加快园区建设步伐，努力把园区打造成为产业集聚发展的重要载体。落实税收、土地、融资、要素保障等中小微企业扶持政策，促进非公经济发展实现新突破。三是大力发展服务业和文化旅游产业。积极发展仓储物流、贸易会展和连锁经营，建成布局合理、功能齐全、水平较高的现代物流产业体系，把陇南打造成甘肃南部商旅物流中心。全面实施电子商务“1333”发展战略，努力把陇南建成全国电商扶贫示范区。深化特色文化大市建设，着力构建“6551”文化产业体系，全面推进文化旅游融合发展。积极融入大九寨旅游经济圈，打造一批精品景区和休闲度假旅游区，创建一批A级景区，不断提升文化旅游产业综合效益。四是不断深化金融改革创新。持续推进“3+1”金融体系建设，发挥好农村产权确权抵押交易服务平台的作用，促进农民增收和农业增效。积极推进中小微企业资金池融资工作，解决中小微企业融资难题，规范做实龙江城乡发展有限公司等投融资主体，更好保障基础设施和重大项目建设资金需求。完善金融服务体系，实现农村资金互助组织贫困村全覆盖。不断扩大金融机构信贷投放规模。运用好落实好棚户区改造贷款和各项惠民信贷政策，充分发挥金融对扶贫攻坚的助推作用。深化“诚信陇南”建设，不断优化金融生态环境。

　　2.坚持改革开放，增强持续发展动力。积极推进供给侧结构性改革，对接用好国家出台的宏观政策、产业政策和社会托底政策，促进经济结构优化升级。进一步转变政府职能，深入推进行政审批制度改革，不断降低制度性交易成本。完成县区政务服务中心和乡镇便民服务大厅规范化建设任务，实现政府权力事项网上规范运行。加快推进公共资源交易电子化、规范化、标准化，充分发挥市场在资源配置中的决定性作用。持续推进财税改革，促进“营改增”和结构性减税政策全面落实。深化行政执法体制改革，逐步开展综合执法。有序推进国有林场改革工作。深化医疗保障、医疗服务、公共卫生、药品供应、监管体制综合改革。稳妥推进户籍制度改革，促进有能力在城镇稳定就业和生活的农业转移人口进城落户。坚持开放发展理念，抢抓国家实施“一带一路”战略的历史机遇，加快建立全方位、多层次开放新格局。进一步深化开放开发，以交通基础设施、文化旅游、特色产业开发、资源利用、生态建设和现代物流、电子商务等新兴产业为重点，谋划实施一批交流合作重点项目，不断深化与川渝、陕南等周边地区特别是邻近市州的务实合作，促进对外交流合作和向南开放不断取得新突破。

　　3.坚持精准发力，确保实现整体脱贫。坚持把扶贫脱贫作为最大的政治任务和第一民生工程，认真贯彻中央扶贫开发工作会议精神，围绕实施“五个一批”工程，聚焦建档立卡贫困村、贫困户和25个特困片区，全面落实省委、省政府“1+17”精准扶贫精准脱贫意见和政策措施，按照“六个精准”和省委“853”精准脱贫管理办法，凝聚帮扶力量，整合扶贫资源，全力打好精准脱贫攻坚战。认真落实精准扶贫精准脱贫各项重点任务，坚持不懈地抓好基础设施建设、资金项目扶持、富民产业培育、自我发展能力提升、易地扶贫搬迁、公共服务保障和生态环境建设等扶贫攻坚工程，着力补齐发展短板。创新生态扶贫、电商扶贫、旅游扶贫等扶贫模式，着力构建专项扶贫、行业扶贫、社会扶贫“三位一体”大扶贫工作格局，鼓励社会资本和民营企业参与扶贫开发，实施“千企帮千村”扶贫行动计划，凝聚全市力量推进脱贫攻坚。促进双联与扶贫深度融合，充分发挥双联单位、驻村帮扶工作队的作用，努力提升扶贫效果。到2020年，9个贫困县区全部摘帽，现行标准下贫困人口全部脱贫。

　　4.坚持项目带动，着力改善发展条件。积极抢抓各类扶持政策叠加的机遇，持续抓好项目争取和实施工作，不断提升投资对经济增长的拉动作用。深入实施“6873”交通突破行动，建成兰渝铁路、渭武高速、徽两高速、武九高速、成州机场和陇南综合客运枢纽等重大交通项目，争取及早开工建设天武铁路和阳九铁路，力争早日建成南下川渝、东进陕西、北上兰州、西连九寨的综合交通运输网络。持续推进国省道和县乡公路升等改造以及县、乡际联网公路建设，抓好运输枢纽站场建设，实现县乡村互联互通。到2020年，实现市内外公路畅通、铁路连通、航路广通三大突破。抓好旅游路规划和建设，实现干线公路与重点旅游景区道路联网。加强水利设施建设，完善防汛、灌溉、供水三大水利体系。加快能源建设，有计划地鼓励发展风电、光电、生物质能源等新能源项目，加大管道天然气建设力度。积极实施“宽带中国”、“互联网+”发展战略，加快信息基础平台和农村通信网络基础建设，全面提升信息化水平。

　　5.坚持统筹发展，提升城乡一体水平。坚持“创新、协调、绿色、开放、共享”发展理念，统筹推进城乡发展，加快形成以城带乡、城乡一体整合发展新格局。做好城市总体规划和基础设施专项规划，统筹布局好空间、规模和产业结构，不断提高城市科学发展水平。加快武都中心城市建设步伐，持续推进各县县城新区建设和旧城改造，抓好小城镇建设，全面实施小城镇综合改革，着力构建以市区为中心、以县城为支撑、以建制镇为骨架的城镇体系。大力推进海绵城市、园林城市试点与建设，推动形成绿色低碳的生活方式和城市建设运营模式，挖掘城市文化内涵，保持良好绿色生态，促进城市发展由外延扩张型向内涵提升式转变。加快城镇基础设施和公共服务设施建设，实施一批城市公共交通、防洪防涝设施项目，抓好综合管廊试点和推广工作，提高城市综合承载能力和防灾避险能力，努力实现生产空间集约高效，生活空间宜居适度，生态空间山清水秀。持续推进生态文明新农村和美丽乡村建设，推动城镇公共服务向农村延伸，努力改善农村人居环境，到2020年，全市建成2200个生态文明新农村。

　　6.坚持生态优先，营造良好人居环境。牢固树立绿色发展理念，坚持保护优先、多措并举，持之以恒推进生态文明建设，努力把陇南建设成为长江上游重要的生态安全屏障。全力实施生态文明示范市、“两江一水”区域综合治理、水生态文明城市建设试点、新一轮退耕还林等重大项目，抓好天然林保护、公益林补偿和长防林建设，加强湖泊、湿地、自然保护区、风景名胜区保护管理工作。加大环境保护力度，积极向国家和省上争取实施有色冶炼、畜禽养殖等污染治理和减排工程，加快城镇生活污水处理和集中供热工程建设，着力解决城乡水污染、城市大气污染、矿区环境污染、农村面源污染、核与辐射等突出问题，实现污染物排放减量化和最小化。实施一批地质灾害防治项目，加强监测预报、灾害预防和应急救援体系建设，完善自然灾害监测预警指挥系统，抓好防汛抗旱减灾工作，不断提升灾害防御能力。

　　7.坚持惠民利民，增进人民群众福祉。始终坚持改善民生，以人为本，着力提升公共服务水平。全面落实创业就业政策措施，加强创业就业服务平台和援助体系建设，开展贫困家庭子女、未升学初高中毕业生、农民工和退役军人免费接受职业培训行动。鼓励贫困乡村吸纳当地农民工参与基础设施和公益设施建设。发挥电子商务、文化旅游等产业的带动作用，着力推进大众创业、万众创新，让创新在全社会蔚然成风。加强社会保障体系建设，加快实现城乡低保、社会保险、救助救济科学化管理。完善社会化养老服务体系，着力提升养老服务水平。加快推进保障性住房和棚户区改造项目建设。深化住房制度改革，把公租房扩大到非户籍人口。坚持不懈地改善民生，每年办成一批民生实事，让广大群众共享改革发展成果。

　　8.坚持协调并进，推进社会和谐进步。优化教育布局，深入推进县域义务教育均衡发展。全面普及学前教育和高中阶段教育，大力发展适用型职业教育，加快发展特殊教育和高等教育，加强教师队伍建设，积极推进素质教育，全面提高教育质量。完成陇南师专升本和陇南卫生职业技术学院创建工作。发挥科技创新在全面创新中的引领作用，强化企业创新主体地位，鼓励支持现有科技单位和科技人员积极开展技术引进和试验、示范、推广，促进科技成果转化应用。加大卫生、体育基础设施建设力度，加快发展体育事业，积极推进民众文体活动和健康陇南建设。扎实推进公共文化服务标准化均等化建设，积极实施文化惠民工程，实现村级文化综合服务中心全覆盖，加强政府采购公共文化服务工作，推动公共文化服务水平迈上新台阶。

　　9.坚持依法行政，推进社会治理创新。加快建设法治政府，健全政府依法决策机制，规范行政执法行为，强化行政行为监督，全面推进政务公开，把法治思维和法治方式贯穿于政府工作全过程，着力提高依法决策、依法行政水平。深入开展法治宣传教育，推动全社会树立法治理念，营造尊法学法守法用法的浓厚氛围。自觉接受人大法律监督和政协民主监督，认真做好建议提案办理工作。加快社会治理体制创新，加强基层社会管理和服务体系建设，全面开展农村社区建设，不断提高基层治理水平。毫不松懈地抓好安全生产、物价监管和食品药品安全监管工作，着力提升公共安全保障水平。深入推进平安陇南建设，努力创造安居乐业的社会环境。

　　10.坚持从严治政，建设人民满意政府。着力提高谋事办事能力，以发展社会事业和解决民生问题为重点，努力解决就医、就学、就业、社会保障、社会治安、安全生产、环境保护等人民群众最关心、最直接、最现实的利益问题，尽心竭力为人民群众提供更多更好的服务；着力提高政府工作效能，大力推广电子政务和网上审批、网上办事，实施重大事项、重点项目清单式管理，建立部门统筹协调机制，形成推动落实的工作合力；着力强化能力作风建设，培养造就一支政治坚定、业务精湛、作风过硬、人民满意的公务员队伍；着力加强政府廉政建设，认真贯彻《中国共产党廉洁自律准则》和《中国共产党纪律处分条例》，严格落实党风廉政建设主体责任和领导干部廉洁自律各项规定，高度重视司法监督、舆论监督和社会公众监督，加强行政监察和审计监督，加大对财政性资金和社会公共资金运行审计力度，强化对扶贫开发、项目建设、公共资源交易等重点工作的监察督查，坚决治理和纠正损害群众利益的突出问题和不正之风，促使公务人员廉洁从政、勤勉尽责，着力提升政府公信力和执行力。深入开展“双查双整”集中行动，不断提高政府管理科学化水平，努力建设人民满意的政府。

　　2016年的目标任务和工作重点

　　今年是实施“十三五”规划和全面建成小康社会进入决胜攻坚的开局之年，经济社会发展任务十分艰巨繁重。做好今年的政府工作，要坚持以党的十八届五中全会精神为指导，认真贯彻落实省委、省政府各项决策部署，深入推进“433”发展战略，打好“十三五”开局战，保持经济社会发展好势头。今年经济社会发展的主要预期目标是：生产总值增长8%，固定资产投资增长10%，规模以上工业增加值增长8.5%，一般公共预算收入增长8%，城镇居民人均可支配收入增长9%，农村居民人均可支配收入增长13%，社会消费品零售总额增长9%，城镇登记失业率控制在4%以内，居民消费价格涨幅控制在3%以内，万元地区生产总值能耗降幅和主要污染物排放指标完成省定目标。

　　围绕确保实现上述目标，重点抓好八个方面的工作：

　　一是加快推进精准扶贫精准脱贫。全面抓好省市精准扶贫政策措施和各项重点任务的落实。深入实施片区扶贫攻坚，加快改善贫困乡村基础设施条件，实施通村硬化路2782公里，通畅率达到100%，行政村饮水安全工程280处，实现贫困村动力电、标准化卫生室全覆盖。加快易地扶贫搬迁进程，优先搬迁生态脆弱区和滑坡体、尾矿区、采空区周边群众，做到应搬尽搬。总结推广两当经验，引导群众通过入股扶贫互助组、专业合作社持续稳定增收。抓好宕昌、徽县互助资金金融改革试点，切实解决贫困户资金难题。充分发挥精准扶贫大数据平台的动态管理作用，抓好市级大数据管理平台工作。深入落实电商扶贫工程，引导贫困户运用“互联网+”参与特色农产品网上销售。加快农村宽带网络建设，扶持培育一批电商扶贫示范网店，切实提高电子商务在贫困乡村的普及程度和应用水平。大力实施“旅游扶贫工程”，启动创建28个旅游扶贫示范村。深入推进科技特派员精准扶贫行动，发挥科技支撑作用。加大劳务输转力度，争取输转劳务67万人。实行扶贫资金全过程监管，确保扶贫资金发挥最大效益。全年减少贫困人口11万人以上。

　　二是加快农业特色产业提质增效。持续开展农业特色产业提质增效年活动，完成200万株核桃、5万株油橄榄、10万株花椒高接换优，完成经济林综合管理670万亩。加快中药材GAP标准化示范基地建设，中药材标准化种植面积达到40万亩。大力推广苹果矮砧密植栽培模式。发展生态放养鸡400万只。新建标准化养殖场（小区）30个。抓好100个市级示范社“十到社”工作，力争合作社覆盖全市所有建档立卡贫困户。设立精准扶贫农业特色产业发展基金，专项扶持农业产业龙头企业、农村合作社等经济组织，提高政府资金利用率。办好特色农产品产销对接洽谈会，积极配合省上办好2016甘肃农业博览会。抓好徽县泥阳省级现代农业科技示范园区、礼县国家级出口食品（苹果）质量安全示范区和宕昌县中药材示范区建设。积极实施“两江一水”菜篮子工程。加快建设以陇南特色农产品交易市场为龙头、乡镇专业市场为依托、各级生产营销协会、合作社为组织形式的市场营销体系。全面开展农村土地承包经营权确权登记颁证工作，稳妥推进农村土地有序流转，陇南市农村产权确权抵押交易平台要上线运行。落实强农惠农政策和各项科技增粮措施，确保粮食总产稳定在98万吨以上。

　　三是促进工业经济稳步增长。加强工业经济运行监测，强化要素保障，促进厂坝有色公司、金徽酒业等重点企业正常生产，加快郭家沟铅锌矿、金徽优质酒酿造技改等项目建设进度，力争年内建成投产。鼓励企业积极推进技术创新，加快淘汰落后产能。加大中小微企业培育和拟入规企业扶持力度，力争更多企业进入规上企业名录，不断扩大规模以上工业经济总量。加快礼县青峰石材等项目建设。进一步加快西成经济开发区、徽县工业集中区建设步伐，创新园区管理运营机制和招商引资方式，发挥项目载体和融资平台的作用，全力推进基础设施建设，着力提升承载能力，不断提高招商引资项目落地率。以“个转企”为契机，积极培育新的工业企业。大力扶持发展非公经济，力争新增非公经济市场主体1万户以上，全市非公经济增加值占生产总值的比重提高1.1个百分点。

　　四是全面提升三产增长速度。加快发展批发零售、住宿餐饮、公路运输、邮政电信等传统服务业，积极扶持发展房地产业，支持信息产业、仓储物流、保险金融、文化创意等现代服务业加快发展。加快推进陇南东盛国际农产商贸物流港、宕昌县哈达铺现代物流园区和成县航空港物流园区建设。

着力抓好电子商务平台建设，把陇南馆打造成我市的电商骨干平台，培育一批电子商务示范企业和示范网店，加快电子商务示范基地、电子商务进农村示范县建设，抓好阿里巴巴陇南产业带“千县万村”、苏宁“农村电商”、京东“千县燎原”等项目建设，依托进出口外贸企业，积极探索跨境电子商务，推进电子商务提质增效。加快官鹅沟景区创建国家5A级旅游景区步伐，抓好官鹅沟国家级地质公园创建工作。大力发展乡村旅游，全力打造28个全国乡村旅游扶贫重点村，加快康县花桥村旅游示范村建设。推进中医药养生保健旅游创新区建设取得突破性进展。办好全省乡村旅游精准扶贫现场会、第二届川陕甘区域旅游合作体创新发展试验区协作会。加快实施大堡子山国家考古遗址公园、长道三国古城、阶州文化城、宕昌县官鹅沟氐羌文化产业园、文县白马人原生态文化旅游区等文化旅游重点项目。加强民俗文化和非物质文化遗产保护传承，抓好历史文化名镇名村和传统村落保护开发，加快推进“乞巧节”和积极参与“蜀道”、“北茶马古道”申报世界文化遗产工作。力争文化产业增加值增长25%以上，全年接待游客数量和旅游综合收入均增长25%，旅游产业增加值占GDP比重达到12.3%。

　　五是深入实施“3341”项目工程。紧盯国家、省上政策和投资导向，积极争取和实施一批重大项目。全力做好协调服务工作，保证兰渝铁路通车安全运营，确保建成成州民用机场，渭武、徽两高速公路完成年度投资计划，力争武九高速公路全线开工建设，礼县至武都公路10月份全面建成通车。抓好国省干线升级改造，建成G567线礼县至成县至康县公路、G212线岭峰至角弓段公路，实施徽县麻沿河至大石碑等普通省道建设。加快实施4A级景区一级一幅旅游路项目，建设4A级景区旅游公路8条共161公里，美丽乡村村组道路354公里。全面推进“6363”水利保障行动，加快民生水利项目建设。完成造林54万亩，建设绿色通道350公里，完成义务植树1100万株，完成天然林保护工程1018.88万亩，重点公益林补偿446.16万亩。加快电网建设，确保酒泉至湖南特高压直流输电工程陇南段建成投运。争取实施国家“农村宽带普遍服务补偿机制试点”和“宽带中国”示范工程，加快行政村和25个特困片区宽带网络覆盖进程。进一步加强招商引资工作，抓好签约项目的协调服务，简化项目审批流程，营造良好的投资环境。实现招商引资到位资金448亿元。

　　六是加快推进城乡一体化建设。完善城乡发展规划，大力推进市区和八县县城建设，提升东江新区、吉石坝新区、钟楼滩新区建设管理水平，抢抓棚户区改造机遇，加快旧城区改造步伐，抓好八县一区城区道路、供排水、供气供热、垃圾污水处理等市政基础设施建设。建成火车站站前广场，加快实施阶州古街、南山公园、滨江体育场等重点项目。统筹抓好礼县、康县及成县小川镇、宕昌县哈达铺镇省级新型城镇化试点，进一步加快9个综合改革试点镇基础设施、公共服务设施和产业发展项目建设，深入推进户籍、土地、投融资等重点改革，示范带动全市新型城镇化进程。全面落实国家、省上促进房地产业持续稳定健康发展的各项政策措施，保持房地产市场健康平稳发展。加快智慧城市建设，推进城市管理现代化和智能化，实现城市管理由粗放管理变为精细管理，不断提高城镇管理水平。加强农村环境综合整治，建设生态文明新农村202个，其中“美丽乡村”示范村42个，环境整洁村160个。

　　七是不断提升财政金融支撑能力。努力培植建设多元财源体系，加强财政收入征管，促进财政收入持续稳定增长。不断优化支出结构，努力压减一般性支出，把保增长、保民生、保稳定、促发展摆在突出位置，进一步完善国库集中支付、部门预算和绩效管理，着力提高资金使用效益。不断完善农村产权确权抵押交易服务平台，全面推进农村产权抵押贷款工作。发挥好市龙江资金池管理有限公司融资职能，为中小微企业和农业龙头企业提供融资担保支持。市信通公司要尽快发挥市级旅游开发融资平台作用。推动各金融机构加大信贷投放力度，确保全年信贷投放增速达到20%以上。进一步做好精准扶贫专项贷款发放工作。稳步发展小额贷款公司、融资性担保公司、农村资金互助组织和村级金融便民服务点，不断健全基层金融服务网络。出台扶持奖励办法，鼓励成长型、创新型企业和农业龙头企业进入“新三板”融资发展，扩大直接融资规模。做好“双联”惠农贷款、草食畜牧业和设施蔬菜、中药材产业等贴息贷款发放工作，为农业特色产业发展提供资金保障。

　　八是推进社会事业全面发展。继续实施全面改善义务教育薄弱学校基本办学条件工程和行政村幼儿园建设项目。推动成县、武都区、两当县县域义务教育均衡发展通过省上和国家评估验收。帮助陇南师专解决升本工作中的实际困难，做好创建陇南护理职业学院相关工作。创新科技服务机制，抓好科技特派员工作，力争专利申请量增长20%以上。加快推进基本公共卫生服务均等化，健全完善全民医保体系，全面实施城乡居民大病保险，建立健全分级诊疗机制和医师多点执业机制，完善贫困乡村医疗卫生体系，不断提高公共卫生和贫困乡村医疗服务水平。稳妥有序实施“一对夫妇生育两个子女”政策。积极开展各级各类卫生创建工作。进一步完善公共文化服务体系，加快村级文化活动室建设，建设乡村舞台746个，完善市县乡村四级全民健身服务网络体系，争取建设30个村级农民体育工程，办好陇南市第八届体育运动会。全面落实就业扶持政策，新增城镇就业2.8万人。进一步做好社会保障和救助工作，落实好城乡最低生活保障政策。抓好保障性住房建设和棚户区改造。实施“七五”普法工作，加快推进依法治市进程。抓好群众信访和矛盾纠纷排查调处工作，整合基层社会治理力量，扎实推进乡镇社会治理办公室建设，加快社会管理信息化建设步伐。加强政法队伍和公共安全专业应急队伍建设，持续推进防控体系建设，有效预防和减少各种不安全不稳定因素，为人民群众提供更好的服务和平安和谐的社会环境。严格落实安全生产监管责任和各项监管措施，加强安全监管队伍建设，加大安全监管执法力度，加强重点行业领域安全管理和隐患排查整治，强化道路交通、建筑工地、特种设备、人员密集场所和大型群众性活动安全管控工作，严防各类重特大安全事故发生。进一步加强食品药品安全监管，认真实施食品药品安全放心工程，落实全程监管制度，深入开展专项整治，严厉打击食品药品违法犯罪行为，切实保障人民群众饮食用药安全。全面落实党的民族宗教政策，加快民族乡村经济社会发展。加强民兵预备役建设和双拥共建工作，持续开展全民国防教育，重视发展老龄、残疾人、档案、红十字会和慈善、公益事业，支持工会、妇联、共青团等群众团体工作，做好农村留守老人、留守儿童工作，维护好职工、妇女儿童和青少年合法权益。

　　今年，要继续办好以下民生实事：（1）实施易地扶贫搬迁贫困人口2万人；（2）完成农村危房改造2万户；（3）完成城市棚户区改造5274套；（4）完成建制村通畅工程2782公里；（5）完成131个贫困村三相动力电建设，实现贫困村全覆盖；（6）建成12个县乡便民市场；（7）提高城乡低保、五保供养水平；（8）免除10万名入园幼儿保教费；（9）解决18万农村人口饮水安全问题。

　　各位代表，“十三五”发展的宏伟蓝图已经绘就。实现整体脱贫和与全国全省一道全面建成小康社会，是各级政府的历史使命和责任担当！让我们在省委、省政府和市委的坚强领导下，紧紧依靠全市人民，同心同德，锐意创新，扎实工作，奋力夺取脱贫攻坚和全面小康社会建设的新胜利，为建设幸福美好新陇南而努力奋斗！
